# Historical ecology of a biological invasion: the interplay of eutrophication and pollution determines time lags in establishment and detection

**DOI:** 10.1007/s10530-017-1634-7

**Published:** 2017-11-24

**Authors:** Paolo G. Albano, Ivo Gallmetzer, Alexandra Haselmair, Adam Tomašových, Michael Stachowitsch, Martin Zuschin

**Affiliations:** 1 Department of Palaeontology, University of Vienna, Althanstrasse 14, 1090 Vienna, Austria; 2 Geological Institute, Slovak Academy of Sciences, Dúbravska Cesta 9, 84005 Bratislava, Slovak Republic; 3 Department of Limnology and Bio-Oceanography, Center of Ecology, University of Vienna, Althanstrasse 14, 1090 Vienna, Austria

**Keywords:** Time lags, Establishment, Hypoxia, Sediment contamination, *Anadara transversa*, Adriatic Sea

## Abstract

Human disturbance modifies selection regimes, depressing native species fitness and enabling the establishment of non-indigenous species with suitable traits. A major impediment to test the effect of disturbance on invasion success is the lack of long-term data on the history of invasions. Here, we overcome this problem and reconstruct the effect of disturbance on the invasion of the bivalve *Anadara transversa* from sediment cores in the Adriatic Sea. We show that (1) the onset of major eutrophication in the 1970s shifted communities towards species tolerating hypoxia, and (2) *A. transversa* was introduced in the 1970s but failed to reach reproductive size until the late 1990s because of metal contamination, resulting in an establishment and detection lag of ~25 years. Subfossil assemblages enabled us to (1) disentangle the distinct stages of invasion, (2) quantify time-lags and (3) finely reconstruct the interaction between environmental factors and the invasion process, showing that while disturbance does promote invasions, a synergism of multiple disturbances can shift selection regimes beyond tolerance limits and induce significant time lags in establishment. The quantification of these time lags enabled us to reject the hypothesis that aquaculture was an initial vector of introduction, making shipping the most probable source.

**Electronic supplementary material:**

The online version of this article (10.1007/s10530-017-1634-7) contains supplementary material, which is available to authorized users.

## Introduction

Anthropogenic disturbance is one of the major drivers of biological invasions (Elton 1958; Cohen and Carlton 1998; Occhipinti-Ambrogi and Savini 2003; Crooks et al. 2011). Severe human-driven disturbances are fast events relative to rates of evolutionary adaptations and can shift selection regimes to conditions different from those in which native species evolved, typically reducing their fitness. In contrast, some non-indigenous species (NIS) may be more adapted to the recipient disturbed conditions, managing to settle and thrive better than competing native species (the “selection regime modification” mechanism, Byers 2002).

Disturbance, however, plays different roles during the multi-stage invasion process. On one hand, it can favor NIS at the introduction stage but, on the other hand, their persistence can be better in low-disturbance conditions (Clark and Johnston 2011). Untangling the selectivity of environmental conditions on invasions thus requires tracing them over longer periods and at fine-scale temporal resolution. Additionally, the interplay of more than one type of disturbance is rarely directly addressed: experiments usually focus on a single causative factor, and detailed observations often begin only after NIS are already established. The result is that little knowledge is available on the factors that influence establishment success.

The study of biological invasions over ecological time scales is hampered by the lack of long-term data on the temporal variation of communities and environmental factors. A unique but under-exploited source of such information is death and subfossil assemblages formed by skeletal remains in sediments (Dietl and Flessa 2011; Kidwell and Tomašových 2013; Dietl et al. 2015; Kosnik and Kowalewski 2016). These assemblages accumulate information on community states over time, and geochronological and paleoecological methods can be used to reconstruct the history of biological invasions and test hypotheses on the mechanisms behind the invasion process.

Here, we test whether the late 20th—early 21st century occurrence of hypoxic events driven by human-induced eutrophication in the northern Adriatic Sea (Justić 1991; Giani et al. 2012) shifted the selection regime in favor of hypoxia-tolerant species and facilitated the establishment of the hypoxia-tolerant invasive bivalve *Anadara transversa* (Say, 1822). This is one of the most invasive species in the Mediterranean Sea (Streftaris and Zenetos 2006). Its native range is eastern and southern North America (Albano et al. 2009); it was first detected in the Mediterranean Sea in Izmir (Turkey) in 1977, in Thessaloniki (Greece) in 1993 and then simultaneously along a 200-km coastline from Venice to Ancona in the northern Adriatic Sea in 2000 (Morello and Solustri 2001; Rinaldi 2001; Mizzan 2002). The sudden occurrence of large populations of large-sized specimens over a 200-km-long coastline cannot be explained by the simultaneous introduction of propagules, suggesting a longer introduction history.

The northern Adriatic is a hotspot of NIS introductions (Ghisotti and Rinaldi 1976; Crocetta 2011, 2012; Occhipinti-Ambrogi et al. 2011) and belongs to the ca. 400 so-called dead zones in the world combining most features that characterize coastal ecosystems sensitive to low oxygen events (Riedel et al. 2012, 2014). It is also subject to riverine input from one of the most anthropized and industrialized European drainage basins and, therefore, it is the ideal setting for testing the relationship between multiple disturbances and invasions.

## Methods

### Study area and sampling

**Fig. 1:**
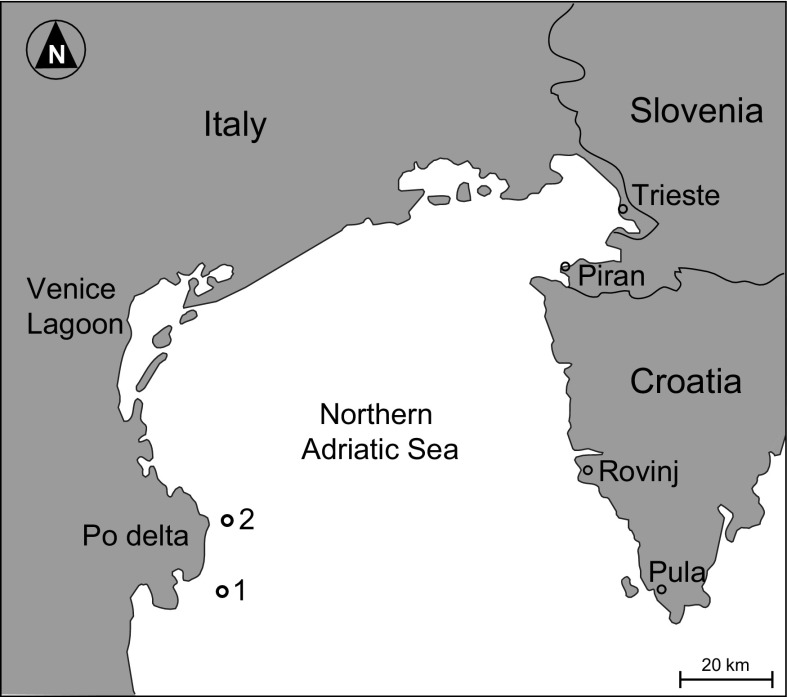
Position of the two stations off the Po river delta

This study is based on sediment cores collected at two stations located off the Po river delta (Fig. 1) in the northern Adriatic Sea: station 1 (44° 43′ 50″ N, 12° 26′ 25″ E, − 21 m) and 2 (44° 50′ 32″ N, 12° 32′ 20″ E, − 23 m). The substrate is muddy at both stations. At each station, two 160-mm-diameter, 150-cm-long cores were taken using an UWITEC^®^ piston corer with hammer action and interchangeable core tube units (Gallmetzer et al. 2016). A third 90-mm-diameter core of the same length was used for sediment dating, grain size and geochemical analyses.

### Sample processing

Core subsamples were sieved with a minimum mesh size of 1 mm. Molluscan shells were identified to species level and counted. Length (i.e., antero-posterior size) was measured in *A. transversa* and four other native bivalve species with abundance > 50 specimens/station: *Nucula nucleus* (Linnaeus, 1758), *Abra alba* (W. Wood, 1802), *Abra nitida* (O.F. Müller, 1776) and *Corbula gibba* (Olivi, 1792). *Abra* data were pooled, given that the two species attain comparable lengths in the Adriatic Sea and share a similar natural history.

**Fig. 2:**
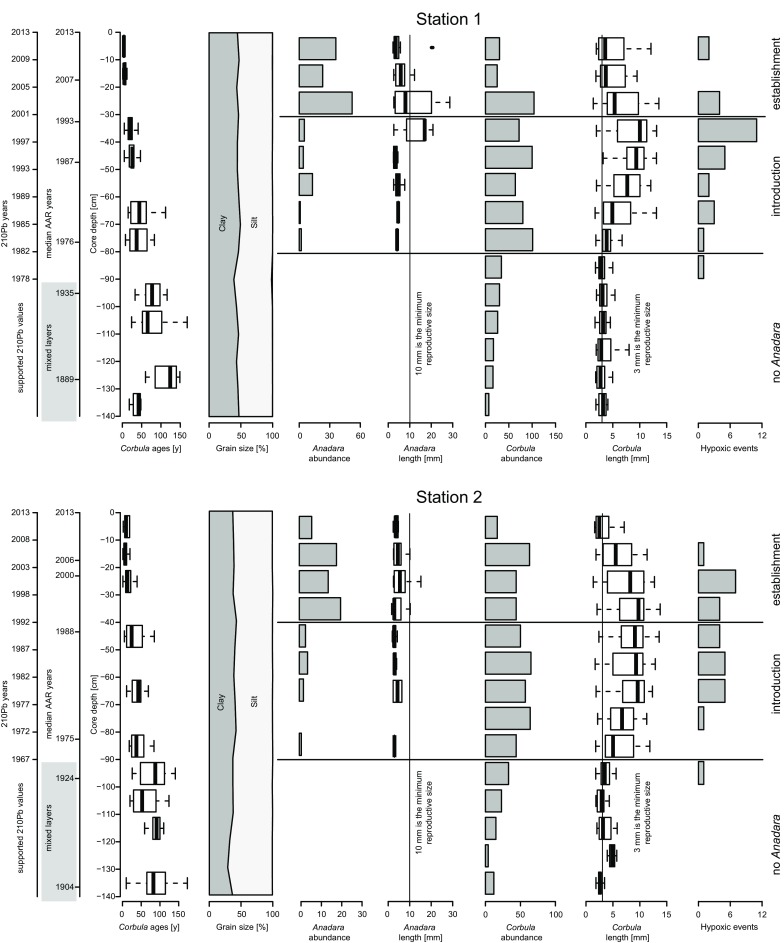
Core stratigraphy; from left to right: sediment and *Corbula gibba* ages (years A.D.), time-averaging quantification (inter-quartile ranges of *Corbula gibba* ages), sediment structure, target species’ abundance (number of individuals) and size, and number of hypoxic events per year after Djakovac et al. (2015) and Justić (1991). ^210^Pb and median shell ages in grey areas suggest sediment mixing. Vertical lines mark the bivalves’ reproductive size (10 and 3 mm, respectively). At both sites, the first occurrence of *Anadara transversa* (Say, 1822) is in the 1970s, 20–30 years earlier than the first record in 2000. Between the 1970s and 2000, the species hardly grew larger than 10 mm and thus did not reproduce. At larger sizes in the late 1990s, reproduction started and population size increased, triggering the first detection in 2000. Since the early 1970s, hypoxic events promoted the hypoxia-tolerant *Corbula gibba* (Olivi, 1792). *A. transversa* is hypoxia-tolerant too, but its introduction was delayed by a peak of metal contamination in the sediments (see Fig. 4)

The reproductive length of *Anadara transversa* was 10 mm in a study conducted in Georgia, USA (Walker and Power 2003). The seawater temperature in the northern Adriatic Sea is slightly lower than in Georgia, potentially causing an increase in reproductive length (Strathmann 1987). Nonetheless, the increases in individual and population sizes are so well marked (Fig. 2) that such variation does not influence our interpretation. Because no information is available for *N. nucleus*, we used data on *N. nitidosa*, which has a similar maximum size and life span. This species is likely sexually mature after 1–2 years; we considered 5 mm as reproductive size because the species attains a size of ca. 3–4 mm after 1 year and then grows 1 mm/year (Rachor and Salzwedel 1976). The first reproductive cycle of *Abra alba* occurs at a length of ca. 8 mm in the Western English Channel (Dauvin and Gentil 1989). The reproductive size of *Corbula gibba* has surprisingly—considering its abundance across the entire northern Adriatic and its hypoxia and disturbance tolerance (Occhipinti-Ambrogi et al. 2002; Holmes and Miller 2006; Nerlović et al. 2011)—not been studied so far. The species attains a length of 3 mm at the age of one year (Jensen 1990). Because of the presumed life-span of 2 to 5 years (Jensen 1990; Hrs-Brenko 2006), we hypothesize a 1-year generation time and thus a reproductive size of 3 mm.

### Geochronology and sediment analyses

The age of sediment layers was quantified using two independent methods: ^210^Pb radiometric sediment dating and radiocarbon calibrated amino acid racemization dating (AAR) of bivalve shells. These two approaches have entirely distinct assumptions and potential biases, making them well suited for cross-validation.

The ^210^Pb analysis was performed at the Low-Level Counting Labor Arsenal at the University of Natural Resources and Life Sciences in Vienna. Activities of ^210^Pb and ^226^Ra were analyzed in 2-cm-thick intervals in the upper 20 cm and in 5-cm-thick intervals between 20 and 40 cm by gamma spectrometry using a High Purity Germanium detector system. We computed apparent sediment-accumulation rates from the slope of the decay in excess ^210^Pb according to the Constant Flux–Constant Sedimentation model (CFCS, Sanchez-Cabeza and Ruiz-Fernández 2012). We avoided the surface mixed layer (SML), which corresponds to the top 20 cm, within which excess ^210^Pb levels remain approximately constant.

Amino acid racemization (AAR) dating is based on the inter-conversion between the two configurations of amino acids: left (L) and right (D) handed (see (Miller et al. 2013) for a recent review of the method). AAR was analyzed on 495 specimens of *C. gibba* selected from 14 increments in the core Po 3 (with midpoints at 2, 6, 10, 14, 18, 22.5, 42.5, 67.5, 82.5, 97.5, 107.5, 112.5, 132.5, and 137.5 cm) and 14 increments in the core Po 4 (with midpoints at 2, 6, 10, 14, 18, 32.5, 47.5, 62.5, 77.5, 92.5, 107.5, 127.5, 132.5, and 152.5 cm). AAR analysis was carried out at Northern Arizona University using reverse-phase high-pressure liquid chromatography (RP-HPLC) and the procedures of Kaufman and Manley (1998). Seventeen specimens were flagged as outliers according to screening criteria outlined by Kosnik and Kaufman (2008), and the 243 shells from station 1 and the 252 shells from station 2 were used for dating. Eleven shells dated by ^14^C and three live-collected shells were used for calibration of amino acid racemization rates to calendar years, using the time-dependent reaction kinetic model (Allen et al. 2013) for Asp D/L, with the initial D/L value estimated from data (TDK1), and lognormal uncertainty (see Tomašových et al. 2017 for details). Sediment ages were plotted against median shell ages to compare chronologies in 10-cm layers. When no shells were dated in a layer, the layer age was estimated by linear interpolation from the layers above and below.

The grain size of 36 samples was analyzed using a Sedigraph for the small fractions (< 63 μm) and sieving for larger fractions. The sediments were classified according to Shepard’s classification (1954). Pollutant concentrations were determined at these core depths: 5, 9, 19, 27, 37, 49, 71, 84, 87, 97, 116, 135 cm and 1, 5, 9, 14, 27, 38, 51, 67, 81, 101, 119 cm at stations 1 and 2, respectively. Geochemical analyses included the content of major (Fe, Al), minor (Mn, P) and trace elements (As, Cd, Cr, Cu, Hg, Ni, Pb, Zn) and persistent organic pollutants (polycyclic aromatic hydrocarbons—PAH, polychlorinated biphenyls—PCB). The detailed analytical protocol is available in Appendix S1.

Raw concentrations were compared to NOAA sediment quality criteria: effects range low (ER-L), representing the threshold level below which effects on benthic organisms rarely occur, and effects range medium (ER-M), above which effects are likely to occur (Burton 2002).

### Hypoxic events and sea temperature

The number of hypoxic events [dissolved oxygen < 2 mg/l, (Diaz and Rosenberg 1995)] per year and the dissolved oxygen at the bottom from 1972 to 2012 were taken from Djakovac et al. (2015). Their stations SJ101 and SJ108 are close to our stations 2 and 1, respectively. Although the surveys of dissolved oxygen concentrations prior to 1972 were collected by variable methods, no hypoxic events were reported between 1911 and 1972 (Justić 1991).

Historical sea surface and bottom temperature data for the Adriatic Sea were obtained from the 1955–2014 Mediterranean Sea physics reanalysis provided by the Copernicus Marine Service Information system (http://marine.copernicus.eu/services-portfolio/access-to-products/?option=com_csw&view=details&product_id=MEDSEA_REANALYSIS_PHY_006_009). The dataset has monthly means of physical parameters with a spatial resolution of 0.06 × 0.06 degrees and 72 water depth levels.

The native range of *A. transversa* was compiled from the literature (Mikkelsen and Bieler 2008). The sea surface temperature along this range was obtained from the Coastal Water Temperature Guide of the NOAA National Centers for Environmental Information (https://www.nodc.noaa.gov/dsdt/cwtg/index.html, last access December 2016).

### Down-core analysis

Down-core variation of abundance, size, pollutants, community composition and structure was assessed focusing on three intervals: (1) layers without *A. transversa* in the lower part of cores; (2) layers in the middle part of cores where *A. transversa* appeared, but was represented by juveniles and abundance was low (introduction stage), and (3) upper layers where *A. transversa* was abundant (establishment stage).

Differences in the whole molluscan community composition in the three stages of invasion were assessed with non-metric multidimensional scaling (NMDS) plots (Kruskal and Wish 1978) and permutational multivariate analysis of variance (PERMANOVA, (McArdle and Anderson 2001; Anderson 2001) based on Bray–Curtis dissimilarities on square-root transformed proportional abundances of molluscan species. Sequential Bonferroni correction was applied to assess significance of pair-wise comparisons (*p* = 0.05). The Similarity Percentage (SIMPER) routine was used to assess the greatest contributors to differences among the three stages (Clarke 1993).

## Results

### Sediment ages and mixing

Sedimentation rates based on excess ^210^Pb profiles below the surface mixed layer (0–20 cm) were 2.3 cm/year and 2.2 cm/year at stations 1 and 2, respectively. Shell median ages show that the top 20 cm correspond to ~ 2006–2013 A.D. and the interval from 20 to 90 cm to the late 20th century after 1950 A.D. with good stratigraphic order. The lower part of the cores corresponds to the early 20th century, with some degree of stratigraphic disorder in the distribution of *C. gibba* ages. ^210^Pb-based estimates of dates assume constant sedimentation rates and tend to be biased upward by bioturbation (Johannessen and Macdonald 2012), whereas AAR estimates of dates can be biased upward or downward by temporally-variable production of shell producers (Tomašových et al. 2015). In addition, mismatches between these methods can be increased by differential mixing of sediment particles varying in size and durability (Barker et al. 2007). Nonetheless, the dates based on both methods highly correlate (Pearson r > 0.8 at both stations, *p* < 0.0001) and show a relatively good correspondence along a one-to-one line.

### Down-core occurrence of *Anadara transversa*

We found 136 and 76 valves of *A. transversa* at station 1 and 2, respectively. At both stations (~ 15 km apart), *A. transversa* first appeared at 80–90 cm, where the median age of *C. gibba* dates back to 1976 (Fig. 2). In the layers immediately above those of the first occurrence, *A. transversa* was scarce (< 5 specimens per layer) and small (rarely exceeding 5 mm). This size corresponds to a post-metamorphic life span of days (Walker and Power 2003). Abundance then increased by an order of magnitude and shell length exceeded the reproductive size (> 10 mm) in layers dating back to the late 1990s (between 30 and 40 cm sediment depth). Above 30–40 cm (since 2000 to present), abundance and size remained at high values.

### Down-core variation in abundance and size of native species

**Fig. 3:**
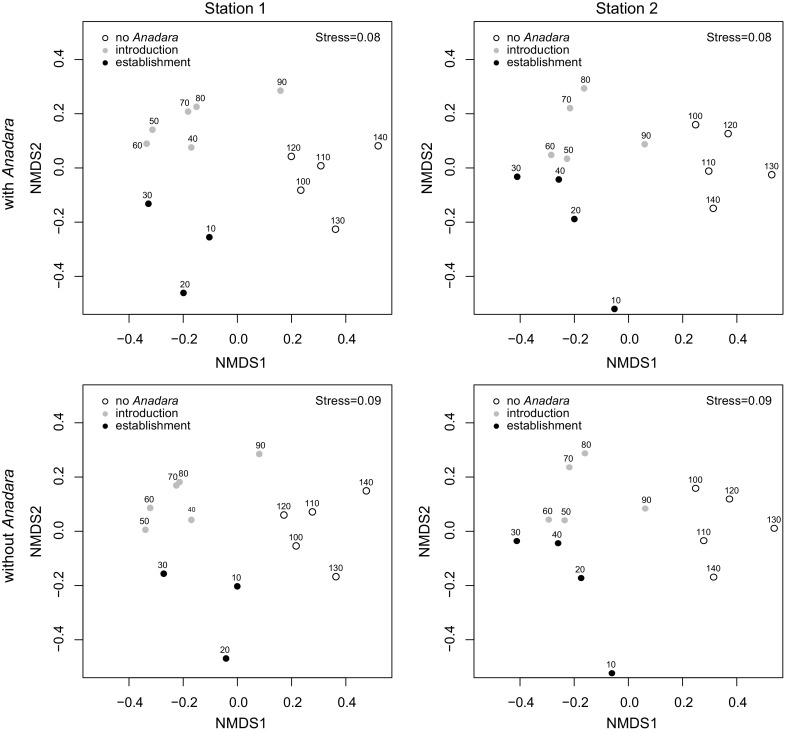
Nonmetric multidimensional scaling plots of down-core assemblages with the full dataset and without the non-indigenous *Anadara transversa* (Say, 1822). The three stages in the introduction of *A. transversa* correspond to distinct states in the molluscan assemblage composition and structure. The identical results with and without *A. transversa* suggest that the assemblages changed irrespective of its introduction

The three stages of the *Anadara* invasion process corresponded to distinct molluscan assemblage states in the multivariate compositional space (Fig. 3; PERMANOVA, global F = 5.7 and 4.5, *p* < 0.001 at both stations, pairwise comparisons in Table S1). The magnitude and significance of differences remain when abundances of *Anadara* are excluded from analyses (Fig. 3), showing that these temporal differences are driven by the variation in relative abundance of some key native species (SIMPER, Table S2).

During the introduction stage, the abundance of *Nucula nucleus* and *Abra* spp. declined markedly compared to the layers before the onset of the introduction. *N. nucleus* and *Abra* spp. partly recovered during the establishment stage of *A. transversa*. *C. gibba* increased considerably during the introduction stage and remained the dominant species also in the upper layers related to the final, establishment stage. The low abundance of *N. nucleus* and *Abra* spp. during the introduction stage was associated with rarity of juveniles, whereas their high abundance during the pre-introduction and establishment stages corresponded with frequent juveniles (Fig. 5). *C. gibba* increased in size during the introduction stage.

### Down-core variation in environmental factors and contaminants

Sediment grain size did not change down-core. The silt plus clay content was 99.7% ± 0.1 and 99.3% ± 0.1 at stations 1 and 2, respectively (Fig. 2). The corresponding percentage of clay were 45.7 ± 0.5 and 37.7 ± 0.8. Since 1955—most of the time-frame captured by the cores—water temperature at the sampled water depths showed no significant trend. In contrast, sea surface temperature has increased, especially since 1980 (Fig. S1), but this variation is well within the boundaries derived from sea surface water temperature along *A. transversa*’s native range (Fig. S2). Since the early 1970s, hypoxic events occurred with increased frequency and intensity until ~2005 (Fig. 2). The number of hypoxic events per layer time interval positively correlates with *C. gibba* abundance and median size. *A. transversa* abundance correlates weakly and its size does not correlate with the number of hypoxic events (Table S3).

**Fig. 4:**
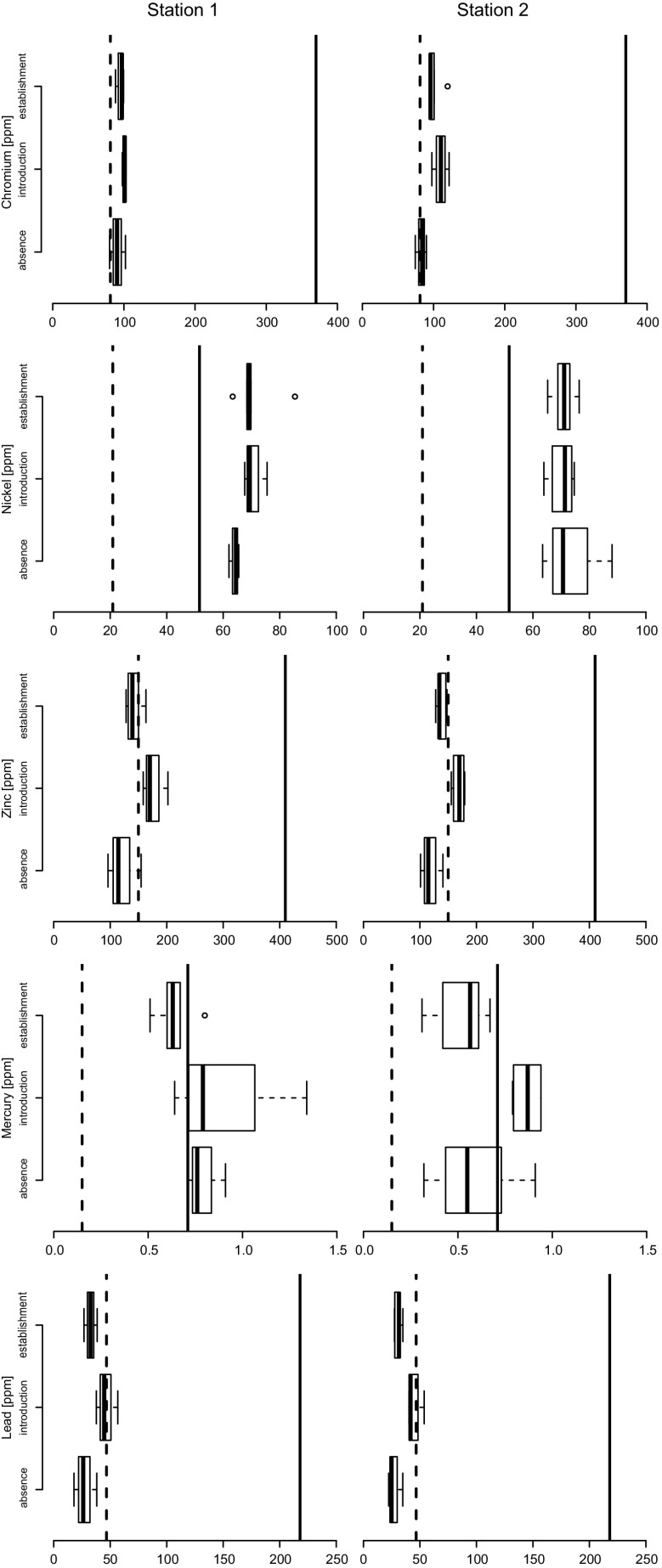
Bar plots of contaminant concentrations. The dashed line is the NOAA ER-L limit (threshold below which effects rarely occur), the solid line is the NOAA ER-M limit (threshold above which effects are likely to occur)

**Fig. 5:**
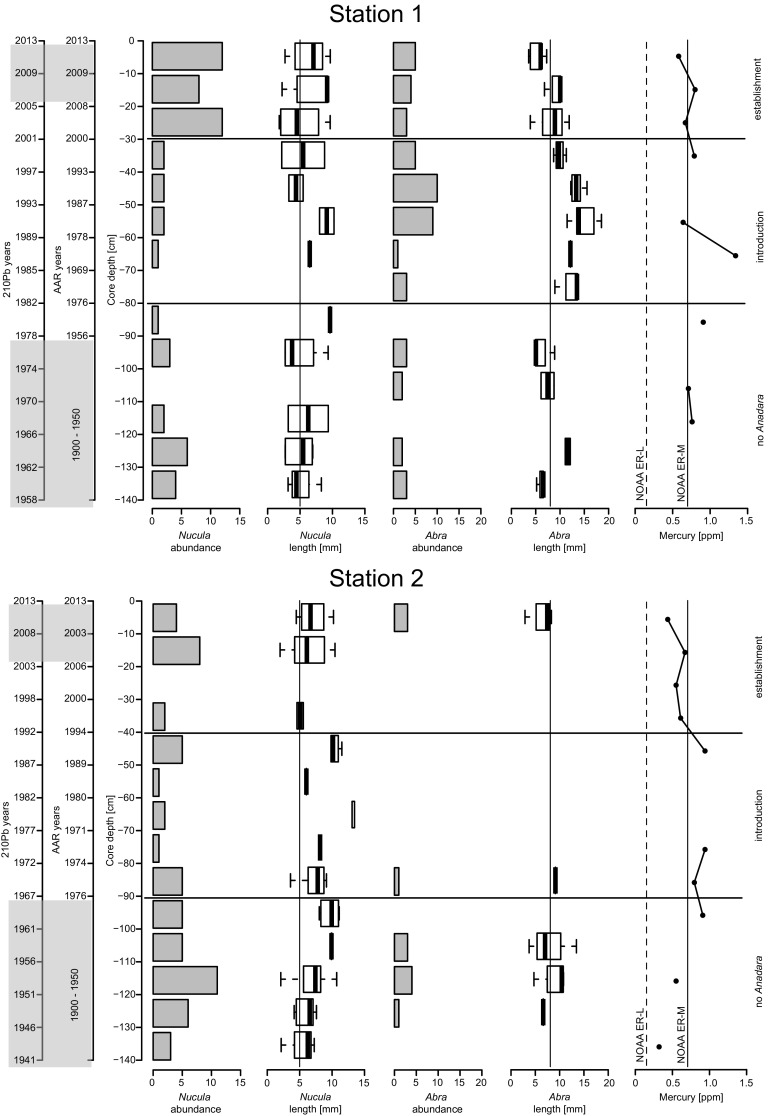
Down-core variation in abundance and shell size of the native bivalves *Nucula nucleus* (Linnaeus, 1758) and *Abra* spp. From left to right: sediment and *Corbula gibba* ages (years A.D.), target species’ abundance (number of individuals) and size, down-core variation of mercury concentration in the sediment. ^210^Pb and AAR Median shell ages in grey areas suggest sediment mixing occurs. Horizontal lines demarcate the three stages of *Anadara transversa* (Say, 1822) invasion. The vertical lines mark the bivalves’ reproductive size (5 and 8 mm, respectively). *N. nucleus* and *Abra* spp. experienced a prolonged lack of juveniles that resulted in a population decline corresponding to the time of increased mercury contamination

During the introduction stage, a distinct peak of sediment contamination by Hg, Ni, Cr, Pb, Zn is detectable (Fig. 4). Especially in the middle part of the cores representing the period from the 1970s to 1990s, Hg concentration was above the NOAA ER-M level (0.7 ppm dw) and reached a maximum of 1.34 ppm dw at 70 cm at station 1 and of 0.94 ppm dw at 80 and 50 cm at station 2 (Fig. 5). Nickel also exceeded the ER-M level of 51.6 ppm dw all along the cores. Chromium and Zn had values only slightly above ER-L, whereas Pb values were below ER-L. The patterns of overall contamination and the absolute values of contaminants are consistent at the two stations.

## Discussion

### Hypoxia and pollution drove the invasion process

Hypoxic events exert a strong effect on benthic assemblages, preferentially affecting larger, long-lived species and promoting hypoxia-tolerant or opportunistic short-lived species (Diaz and Rosenberg 1995). The assemblages off the Po delta responded to the increased occurrence and severity of hypoxia starting in the 1970s (Justić 1991), profoundly changing their composition (Fig. 3, upper panels) as also observed elsewhere in the northern Adriatic (Crema et al. 1991; Chiantore et al. 2001; Occhipinti-Ambrogi et al. 2005; Kowalewski et al. 2015; Gallmetzer et al. 2017). The hypoxia-tolerant *Corbula gibba* thrived: its abundance and median size increased in the early 1970s, reaching two to three times the values typical of the early 20th century. Such changes were not driven by the introduction of *Anadara transversa*, because the NMDS plots are virtually identical if the species is removed from the dataset (Fig. 3, lower panels).

This new selection regime, unprecedented since the early 20th century, should have facilitated the establishment of hypoxia-tolerant non-native species such as *A. transversa* because Anadarinae possess hemoglobin and erythrocytes in the blood and are thus resistant to hypoxia (Broom 1985). However, since its arrival in the mid-1970s and for the following 25 years, *A. transversa* failed to establish notwithstanding continuous propagule input, as evidenced by the constant presence of juvenile shells in the sediments.

We found that the introduction stage was also marked by the rarity of juveniles of locally abundant species such as *Nucula nucleus* and *Abra* spp. in death assemblages, suggesting that environmental factors negatively affected their reproduction or recruitment. Although hypoxia can affect bivalve larval settlement (Weis 2014), this is unlikely to occur in both *N. nucleus* and *Abra* spp. because they spawn in late autumn and winter, i.e., after the occurrence of seasonal hypoxic crises (Chardy et al. 1984; Dauvin and Gentil 1989).

Instead, during the introduction stage we detected a peak of sediment contamination by several metals with a particular prevalence of Hg, whose concentrations were above NOAA ER-L, i.e., adverse effects are likely to occur. Mercury negatively affects bivalve burrowing behavior at concentrations of 0.46 ppm (McGreer 1979), but its effects may be more severe for the settlement and survival of larvae and juveniles, the most sensitive life stages, and affect recruitment (McGreer 1982; Bryan and Langston 1992; Boening 2000). Other metals can also negatively affect bivalves. For example, Zn and Cu concentrations lower than those we detected slowed burial in the tellinid *Macomona liliana* (Roper et al. 1995). Metals affect embryonic development, leading to abnormal embryos, and larvae may be even more sensitive than embryonic stages of the same organism because embryos are protected by an outer membrane that may reduce contaminant uptake (Weis 2014). This contamination may explain why *A. transversa* larvae, likely introduced with ballast water from distant parent populations, were affected: larval exposures to contaminants can impair settlement in the benthic environment and/or delay physiological development as juveniles or adults. This would explain the occurrence of only very young *A. transversa* during the introduction phase. Therefore, in contrast to previous findings where pollution preferentially affected native species (Piola and Johnston 2008; Varó et al. 2015), this contamination prevented the establishment of *A. transversa*. Sediment contamination may therefore interfere with the expectation that hypoxic events enhance the success of invasive species in marine and estuarine ecosystems (Byers 2000; Jewett et al. 2005; Lagos et al. 2017). Then, the decline of metal contamination in the early 2000s generated a new selection regime in which hypoxic events and other disturbances unrelated to metal pollution, including changes in the Po River flow rate and temporary reductions in eutrophication pressure (Occhipinti-Ambrogi et al. 2005; Mozetič et al. 2010; Djakovac et al. 2012), filtered native and non-indigenous species. The hypoxia-tolerant *A. transversa* thrived, finally reached reproductive size and established large self-sustaining populations, triggering the first detection.

Our study provides strong evidence that in ecosystems subject to multiple disturbances, invasion success can be difficult to predict because it is the outcome of the complex interplay between multiple selection regimes. Disturbance can shift such regimes even beyond non-indigenous species tolerance limits, causing significant time lags in establishment.

### Quantification of time lags enables proper vector identification

Correct placement in time of the arrival and establishment of *A. transversa* enables the reliable identification of the dispersal vector. Juvenile *A. transversa* can secrete byssus and attach to hard objects such as rocks and shells (Solustri et al. 2003). Therefore, the transfer of mussels and other aquaculture products from Turkey to northern Adriatic countries could have been an effective primary vector of dispersal. Based on our reconstructed history of the *A. transversa* introduction, we reject this hypothesis because mussel farming started in Turkey only in the 1990s (FAO 2005), ca. 20 years after the first introduction in the Adriatic Sea. Importantly, this hypothesis could not have been rejected assuming the year of first record (the 2000) as the year of first introduction. Most probably, the primary vector of dispersal is shipping, either as larvae in ballast water or as fouling. The analysis of ship traffic time series from 2000 to 2015 of the port of Ravenna (a few kilometers from the study site) shows that two to three hundred ships per year came from Turkey (6–9% of total traffic), of which 40% came from Izmir.

### Lag times in first detection

Because most non-indigenous species (NIS) introductions in the sea are unintentional, the initial invasion stages (Blackburn et al. 2011) are rarely observed and the timing of first introduction is generally not known. Occasionally, the first arrival of NIS is backdated based on findings of specimens of known collection date in museums or private collections [e.g., a time lag of 25 years was quantified for the lessepsian fish *Fistularia commersonii* (Bariche et al. 2014) and of 150 years for the brackish water bivalve *Mytilopsis leucophaeata* in Great Britain (Oliver 2015)]. Although these findings are precious for the reconstruction of invasion history, they are based on serendipitous events and not on a reproducible method, hindering any large-scale assessment of detection lags, their causes and consequences.

Here we used the subfossil record preserved in sediment cores to show that the first occurrence of *A. transversa* in the northern Adriatic Sea dates back to the 1970s. This is a time lag in first detection of about 25 years, almost double its history after detection. This lag corresponds to the lengthy duration of its introduction phase: the species was then detected as soon as it established. The lack of appreciation of time lags causes the underestimation of the potential consequences of a particular invasion. This, in turn, can impair accurate risk assessment because the time since introduction is the best predictor of the global geographic range of marine invaders (Crooks et al. 2011; Byers et al. 2015). Finally, underestimated introduction modalities delay action by decision makers in defining priorities and setting prevention measures (Belmaker et al. 2009).

Our data show that for ~25 years, *A. transversa* remained very small (median size ≤ 5 mm) and with very limited population size. Combined, these two factors hampered its detection. Low detectability has certainly been coupled with a limited specific monitoring effort. Indeed, the first record occurred in the context of surveys with hydraulic dredges to evaluate stocks of edible clams (Morello and Solustri 2001), where the minimum mesh size used was 6 mm, but most often 12 or even 40 mm (Morello et al. 2004). The first recorded specimens were between 10 and 31 mm in size (median: 21 mm). Ultimately, the large size of the specimens also facilitated species identification by non-specialists.

### Beyond the time impediment

The differential role of disturbance on the early versus late stages of invasions is rarely captured along the entire introduction history of an invader (Clark and Johnston 2011; Clark et al. 2013), and retrieving data on pre-disturbance environmental conditions is difficult (Byers 2002). Our study demonstrates that death assemblages enable reconstructing the invasion history and the time variation of environmental variables at decadal time scales and with almost yearly temporal resolution. Indeed, we were able to identify disturbance-driven shifts in selection regimes, the responses of native and non-indigenous species and the underlying biological mechanisms; we also quantified time lags in first detection and establishment. We suggest that death assemblages will prove precious also in addressing other fundamental issues in invasion ecology, such as disclosing the status of cryptogenic species [whose status as native or non-native is not known (Carlton 1996)] and discriminating pseudoindigenes [introduced species which are mistakenly considered as natives (Carlton 2009)] from true native species (Van Leeuwen et al. 2005, 2008; Coffey et al. 2011).

Death assemblages are a still under-exploited but extremely promising source of information to provide a new perspective on biological invasions and their histories, guaranteeing standardized approaches and quantitative measurements.

## Electronic supplementary material

Below is the link to the electronic supplementary material.

Supplementary material 1 (DOCX 89 kb)
