# Supplementary material for: Historical ecology of a biological invasion: the interplay of eutrophication and pollution determines time lags in establishment and detection
Source: Biol Invasions. 2017 Nov 24;20(6):1417–30. doi: 10.1007/s10530-017-1634-7 (PMC5959955; doi:10.1007/s10530-017-1634-7)
Supplement: Supplementary file 1 — Supplementary material 1 (DOCX 89 kb) [file 10530_2017_1634_MOESM1_ESM.docx]

**Historical ecology of a biological invasion: the interplay of eutrophication and pollution determines time lags in establishment and detection**

**Supporting information**

Paolo G. Albano, Ivo Gallmetzer, Alexandra Haselmair, Adam Tomašových, Michael Stachowitsch, Martin Zuschin

# Index

Index 1

1 Detailed procedure for the analysis of heavy metals in sediments 2

2 Additional figures 4

3 Additional tables 5

4 References 15

# Detailed procedure for the analysis of heavy metals in sediments

To analyze elemental concentrations, each sediment sample was gently squeezed to break down aggregates and screened through a PE sieve to remove particles bigger than 1 mm. A part of the screened sediment was dried in an oven at 105 °C until reaching a constant weight (to measure water content). The dried sediment was ground to powder using an agate mortar and pestle before further analyzing the contents of heavy metals and As. The sample (about 0.4 g d.w.) was digested with 8 ml HNO_3_ in a microwave oven (Multiwave 3000, Anton Paar, Austria). The digested material was left to cool at room temperature and then filtered through a 0.45 μm nitrocellulose membrane filter. The filtered digestates were diluted with distilled deionized water to 40 ml in a volumetric flask (USEPA, 1994a). The concentrations of the elements (Al, As, Cd, Cr, Cu, Fe, Mn, Ni, Pb, Zn) were determined by inductively coupled plasma atomic emission spectrometry (ICP-AES) (Optima 2100DV, Perkin Elmer, USA) (USEPA, 1994b). Mercury analyses were carried out using atomic absorption spectrophotometry with cold vapor (Analyst 100, Perkin Elmer, USA) (USEPA, 1976).

The quality acceptance protocols required that one blank sample or one certified reference material (BCR-277r estuarine sediment, Community Bureau of Reference) were digested and analyzed with each batch of fifteen samples. The blank results indicated that the analytical procedure was free from contamination because the concentrations of all metals were below the respective method detection limits. Mean recovery from the certified material ranged between 84% (Zn) and 103% (Hg), except for Al (40%) because the extraction method was not strong enough to break crystalline aluminosilicates. The analytical precision, determined using five replicates of homogenized samples, was estimated to be better than 10% for all elements. Calibration for ICP-AES and AAS analysis was achieved with prepared external standards via the standard curve approach. Full calibration was performed after every set of 48 samples. The method detection limit for element analysis was defined as 3 times the standard deviation of 10 blank measurements.

To analyze the concentrations of persistent organic pollutants, sediment samples were thoroughly mixed, sieved through a 1 mm mesh to remove any debris, and subsequently air dried in the dark at room temperature for 48 h on hexane rinsed aluminum foil. The dry samples were finely ground in an agate mortar. The extraction was performed using a Microwave Sample Preparation System (Multiwave 3000, Anton Paar Graz, Austria), in accordance with the EPA recommendation (method 3546). Two grams of dried sediments were weighed into lined microwave extraction vessels. Then, a 25 ml 1:1 acetone/hexane solvent mixture was added. The vessels were then assembled as instructed by the manufacturer and the extraction was conducted during 15 min at 110 °C and 6-10 bars. At the end of the oven program, vessels were cooled to room temperature and the extracts were filtered and rinsed with the same solvent mixture.

The samples were concentrated in a rotating evaporator (Rotavapor-R Buchi, CH), and the sulphur compounds were removed by soaking the extracts with activated copper powder. Purification and fractionation were performed by eluting extracts through chromatography glass columns packed with Silica gel/Alumina/Florisil (4+4+1 gr). The first fraction, containing PCBs, was eluted with 25ml of n-hexane, whereas the second fraction, containing the PAHs, was eluted with 30 ml of 8:2 n-hexane/methylene chloride solvent mixture (Fossato et al., 1996, 1998). After concentration with a rotary evaporator, the samples were ready for the instrumental analysis.

The identification of PAHs and PCBs was based on matching retention time, and the quantification was obtained from calibration curves established for each compound by analyzing four external standards. Average determination coefficients R2 of the calibration curves exceeded 0.99 for both PAH and PCB, and the relative standard deviations of the calibration factors were always less than 20% (average 10%). The detection limits were 0.05-0.1 ng g-1 and 0.05 ng g-1 for PAHs and PCBs, respectively. Blanks were run for the entire procedure. Recovery and accuracy were validated with IAEA-417 and IAEA-159 sediment sample certified reference materials. Laboratory methods were also validated by intercalibration activities (IAEA, 2001, 2007, 2012).

# Additional figures

**Fig. S1**. Sea surface temperature (red) and bottom temperature at -20 m (blue) at site 44.75° N 12.4375° E. Data from the 1955-2014 Mediterranean Sea physics reanalysis provided by the Copernicus Marine Service Information system and produced by the National Institute of Geophysics and Volcanology, Euro-Mediterranean Center on Climate Change (http://marine.copernicus.eu/services-portfolio/access-to-products/?option=com_csw&view=details&product_id=MEDSEA_REANALYSIS_PHY_006_009, last access March 2016)

**Figure S2**. Annual variation of sea surface temperature at the northernmost limit of *Anadara transversa* native range (Massachusetts) and at its southernmost limit (Texas). Median sea surface temperature and confidence intervals at site 44.75° N 12.4375° E off the Po delta are shown to demonstrate that they are well within the temperature range of the native distribution

# Additional tables

**Table S1**. Details of PERMANOVA model to test for differences in community structure and composition between different stages of the *Anadara transversa* invasion process.

|  | **Degrees of freedom** | **Sum of squares** | **Mean squares** | **F** | **p** |
| --- | --- | --- | --- | --- | --- |
| **Station 1 – Global test** | | | | | |
| Assemblage | 2 | 0.54 | 0.27 | 5.72 | 0.001 |
| Residuals | 11 | 0.52 | 0.05 |  |  |
| Total | 13 | 1.06 |  |  |  |
| **Station 1 – Stage without Anadara vs introduction** | | | | | |
| Assemblage | 1 | 0.33 | 0.33 | 7.08 | 0.002 |
| Residuals | 9 | 0.42 | 0.05 |  |  |
| Total | 10 | 0.75 |  |  |  |
| **Station 1 – Stage without Anadara vs establishment** | | | | | |
| Assemblage | 1 | 0.27 | 0.27 | 4.89 | 0.012 |
| Residuals | 7 | 0.39 | 0.06 |  |  |
| Total | 8 | 0.66 |  |  |  |
| **Station 1 – Introduction vs establishment** | | | | | |
| Assemblage | 1 | 0.19 | 0.19 | 4.87 | 0.019 |
| Residuals | 6 | 0.24 | 0.39 |  |  |
| Total | 7 | 0.43 |  |  |  |
| **Station 2 – Global test** | | | | | |
| Assemblage | 2 | 0.48 | 0.24 | 4.47 | 0.001 |
| Residuals | 11 | 0.59 | 0.05 |  |  |
| Total | 13 | 1.08 |  |  |  |
| **Station 2 – Stage without Anadara vs introduction** | | | | | |
| Assemblage | 1 | 0.27 | 0.27 | 5.14 | 0.009 |
| Residuals | 8 | 0.42 | 0.05 |  |  |
| Total | 9 | 0.68 |  |  |  |
| **Station 2 – Stage without Anadara vs establishment** | | | | | |
| Assemblage | 1 | 0.32 | 0.32 | 5.73 | 0.009 |
| Residuals | 7 | 0.39 | 0.06 |  |  |
| Total | 8 | 0.71 |  |  |  |
| **Station 2 – Introduction vs establishment** | | | | | |
| Assemblage | 1 | 0.13 | 0.13 | 2.45 | 0.007 |
| Residuals | 7 | 0.38 | 0.05 |  |  |
| Total | 8 | 0.51 |  |  |  |

**Table S2**. Results of the SIMPER routine, highlighting the relative contribution of species to differences between groups.

| **Species** | **Average contribution to overall dissimilarity** | **SD of contribution to overall dissimilarity** | **Average to SD ratio** | **Average abundance group A** | **Average abundance group B** | **Cumulative contribution to dissimilarity** |  |
| --- | --- | --- | --- | --- | --- | --- | --- |
| **Station 1 – Introduction stage (group A) vs stage without *Anadara* (group B)** | | | | | | | |
| *Turritella communis* | 0.035 | 0.011 | 3.066 | 0.190 | 0.441 | 0.080 |  |
| *Nassarius pygmaeus* | 0.025 | 0.011 | 2.216 | 0.316 | 0.135 | 0.137 |  |
| *Corbula gibba* | 0.023 | 0.008 | 3.030 | 0.735 | 0.569 | 0.190 |  |
| *Kurtiella bidentata* | 0.019 | 0.010 | 1.968 | 0.297 | 0.428 | 0.234 |  |
| *Nucula cf nuclaeus* | 0.019 | 0.006 | 3.002 | 0.086 | 0.222 | 0.277 |  |
| *Bittium submamillatum* | 0.019 | 0.011 | 1.746 | 0.167 | 0.038 | 0.319 |  |
| *Aporrhais pespelecani* | 0.015 | 0.007 | 2.135 | 0.125 | 0.019 | 0.354 |  |
| *Phaxas adriaticus* | 0.013 | 0.007 | 1.860 | 0.011 | 0.103 | 0.384 |  |
| *Anadara transversa* | 0.013 | 0.006 | 2.269 | 0.093 | 0.000 | 0.414 |  |
| *Polititapes* cf *rhomboides* | 0.012 | 0.006 | 2.055 | 0.099 | 0.019 | 0.442 |  |
| *Saxicavella jeffreysi* | 0.011 | 0.008 | 1.371 | 0.103 | 0.134 | 0.467 |  |
| *Coracuta obliquata* | 0.011 | 0.008 | 1.453 | 0.016 | 0.091 | 0.492 |  |
| *Flexopecten glaber* | 0.011 | 0.007 | 1.572 | 0.120 | 0.045 | 0.517 |  |
| *Aequipecten opercularis* | 0.010 | 0.007 | 1.522 | 0.139 | 0.176 | 0.541 |  |
| *Acanthocardia paucicostata* | 0.010 | 0.004 | 2.180 | 0.066 | 0.113 | 0.563 |  |
| *Abra alba* | 0.009 | 0.006 | 1.476 | 0.074 | 0.059 | 0.584 |  |
| *Musculus subpictus* | 0.008 | 0.005 | 1.565 | 0.062 | 0.015 | 0.602 |  |
| *Calyptraea chinensis* | 0.008 | 0.005 | 1.679 | 0.051 | 0.055 | 0.620 |  |
| *Hiatella arctica* | 0.008 | 0.007 | 1.151 | 0.053 | 0.019 | 0.637 |  |
| *Cylichna cylindracea* | 0.007 | 0.005 | 1.396 | 0.039 | 0.066 | 0.654 |  |
| *Thyasira biplicata* | 0.007 | 0.006 | 1.141 | 0.053 | 0.015 | 0.671 |  |
| *Moerella* cf *distorta* | 0.007 | 0.004 | 1.822 | 0.052 | 0.000 | 0.687 |  |
| *Abra nitida* | 0.007 | 0.005 | 1.313 | 0.095 | 0.078 | 0.703 |  |
| *Spisula subtruncata* | 0.007 | 0.004 | 1.660 | 0.051 | 0.013 | 0.718 |  |
| *Antalis inaequicostata* | 0.007 | 0.007 | 0.990 | 0.017 | 0.046 | 0.733 |  |
| *Hyala vitrea* | 0.006 | 0.005 | 1.165 | 0.117 | 0.094 | 0.748 |  |
| *Eulima glabra* | 0.006 | 0.005 | 1.178 | 0.024 | 0.044 | 0.762 |  |
| *Anomia ephippium* | 0.006 | 0.006 | 1.047 | 0.024 | 0.034 | 0.775 |  |
| *Philine quadripartita* | 0.005 | 0.006 | 0.815 | 0.017 | 0.030 | 0.787 |  |
| *Pitar rudis* | 0.005 | 0.004 | 1.420 | 0.072 | 0.079 | 0.798 |  |
| *Mangelia costulata* | 0.005 | 0.005 | 0.980 | 0.028 | 0.025 | 0.809 |  |
| *Philine scabra* | 0.005 | 0.006 | 0.796 | 0.035 | 0.000 | 0.820 |  |
| *Odostomia* spp | 0.005 | 0.007 | 0.672 | 0.000 | 0.034 | 0.831 |  |
| *Pecten jacobaeus* | 0.004 | 0.006 | 0.682 | 0.000 | 0.031 | 0.840 |  |
| *Mysia undata* | 0.004 | 0.005 | 0.879 | 0.027 | 0.012 | 0.850 |  |
| *Lentidium mediterraneum* | 0.004 | 0.005 | 0.840 | 0.012 | 0.025 | 0.859 |  |
| *Fusinus rostratus* | 0.004 | 0.006 | 0.692 | 0.000 | 0.028 | 0.868 |  |
| *Parvicardium minimum* | 0.003 | 0.006 | 0.635 | 0.011 | 0.017 | 0.876 |  |
| *Nassarius lima* | 0.003 | 0.005 | 0.694 | 0.000 | 0.025 | 0.884 |  |
| *Odostomia* cf *eulimoides* | 0.003 | 0.005 | 0.646 | 0.017 | 0.013 | 0.892 |  |
| *Ostrea* spp | 0.003 | 0.005 | 0.655 | 0.012 | 0.015 | 0.899 |  |
| *Monia patelliformes* | 0.003 | 0.004 | 0.802 | 0.023 | 0.000 | 0.906 |  |
| *Mimachlamys varia* | 0.003 | 0.006 | 0.440 | 0.000 | 0.020 | 0.912 |  |
| *Euspira macilenta* | 0.003 | 0.006 | 0.440 | 0.000 | 0.020 | 0.919 |  |
| *Hexaplex trunculus juv* | 0.003 | 0.006 | 0.440 | 0.000 | 0.020 | 0.925 |  |
| *Hydrobia* sp 1 cf *acuta* | 0.003 | 0.006 | 0.440 | 0.000 | 0.017 | 0.931 |  |
| *Akera bullata* | 0.003 | 0.006 | 0.440 | 0.000 | 0.017 | 0.937 |  |
| *Nucula sulcata* | 0.002 | 0.005 | 0.440 | 0.000 | 0.019 | 0.942 |  |
| *Epitonium muricatum* | 0.002 | 0.005 | 0.440 | 0.000 | 0.015 | 0.947 |  |
| *Fustiaria rubescens* | 0.002 | 0.004 | 0.440 | 0.000 | 0.013 | 0.951 |  |
| *Timoclea ovata* | 0.002 | 0.004 | 0.491 | 0.012 | 0.000 | 0.955 |  |
| *Bivalvia* indet | 0.002 | 0.004 | 0.491 | 0.012 | 0.000 | 0.959 |  |
| Land snail 1 | 0.002 | 0.004 | 0.491 | 0.012 | 0.000 | 0.963 |  |
| *Cerastoderma edule* | 0.002 | 0.004 | 0.440 | 0.000 | 0.013 | 0.967 |  |
| *Mangelia attenuata* | 0.002 | 0.004 | 0.440 | 0.000 | 0.013 | 0.971 |  |
| *Acteon tornatilis* | 0.002 | 0.004 | 0.440 | 0.000 | 0.013 | 0.974 |  |
| *Gastropoda indet* | 0.002 | 0.004 | 0.440 | 0.000 | 0.013 | 0.978 |  |
| *Papillicardium papillosum* | 0.002 | 0.003 | 0.491 | 0.012 | 0.000 | 0.982 |  |
| *Nassarius reticulatus* | 0.002 | 0.003 | 0.491 | 0.012 | 0.000 | 0.986 |  |
| *Ringicula conformis* | 0.002 | 0.003 | 0.491 | 0.012 | 0.000 | 0.990 |  |
| *Anadara inaequivalvis* | 0.002 | 0.003 | 0.491 | 0.011 | 0.000 | 0.993 |  |
| *Pectinidae indet* | 0.002 | 0.003 | 0.491 | 0.011 | 0.000 | 0.997 |  |
| *Euspira nitida* | 0.002 | 0.003 | 0.491 | 0.011 | 0.000 | 1.000 |  |
| **Station 1 – Establishment stage (group A) vs stage without *Anadara* (group B)** | | | | | | | |
| *Anadara transversa* | 0.051 | 0.006 | 8.082 | 0.362 | 0.000 | 0.110 |  |
| *Turritella communis* | 0.033 | 0.012 | 2.830 | 0.206 | 0.441 | 0.181 |  |
| *Kurtiella bidentata* | 0.030 | 0.010 | 2.855 | 0.215 | 0.428 | 0.245 |  |
| *Aequipecten opercularis* | 0.020 | 0.008 | 2.347 | 0.033 | 0.176 | 0.288 |  |
| *Saxicavella jeffreysi* | 0.017 | 0.010 | 1.746 | 0.019 | 0.134 | 0.324 |  |
| *Moerella* cf *distorta* | 0.017 | 0.003 | 6.101 | 0.119 | 0.000 | 0.360 |  |
| *Corbula gibba* | 0.016 | 0.010 | 1.571 | 0.684 | 0.569 | 0.395 |  |
| *Flexopecten glaber* | 0.015 | 0.010 | 1.412 | 0.145 | 0.045 | 0.427 |  |
| *Coracuta obliquata* | 0.013 | 0.008 | 1.668 | 0.000 | 0.091 | 0.454 |  |
| *Abra nitida* | 0.012 | 0.008 | 1.507 | 0.166 | 0.078 | 0.481 |  |
| *Phaxas adriaticus* | 0.012 | 0.007 | 1.681 | 0.028 | 0.103 | 0.506 |  |
| *Pitar rudis* | 0.012 | 0.009 | 1.355 | 0.155 | 0.079 | 0.531 |  |
| *Nassarius pygmaeus* | 0.011 | 0.008 | 1.299 | 0.104 | 0.135 | 0.554 |  |
| *Cylichna cylindracea* | 0.011 | 0.008 | 1.281 | 0.136 | 0.066 | 0.578 |  |
| *Acanthocardia paucicostata* | 0.010 | 0.007 | 1.382 | 0.066 | 0.113 | 0.599 |  |
| *Nucula* cf *nuclaeus* | 0.010 | 0.007 | 1.502 | 0.269 | 0.222 | 0.621 |  |
| *Abra alba* | 0.010 | 0.008 | 1.259 | 0.074 | 0.059 | 0.642 |  |
| *Hyala vitrea* | 0.009 | 0.009 | 1.067 | 0.146 | 0.094 | 0.662 |  |
| *Calyptraea chinensis* | 0.008 | 0.006 | 1.329 | 0.060 | 0.055 | 0.679 |  |
| *Thyasira biplicata* | 0.008 | 0.006 | 1.259 | 0.060 | 0.015 | 0.696 |  |
| *Antalis inaequicostata* | 0.008 | 0.006 | 1.265 | 0.061 | 0.046 | 0.712 |  |
| *Polititapes* cf *rhomboides* | 0.007 | 0.005 | 1.345 | 0.047 | 0.019 | 0.727 |  |
| *Anomia ephippium* | 0.006 | 0.007 | 0.938 | 0.028 | 0.034 | 0.741 |  |
| *Eulima glabra* | 0.006 | 0.005 | 1.113 | 0.019 | 0.044 | 0.754 |  |
| *Nassarius lima* | 0.006 | 0.006 | 0.951 | 0.033 | 0.025 | 0.767 |  |
| *Mangelia costulata* | 0.006 | 0.006 | 0.949 | 0.033 | 0.025 | 0.779 |  |
| *Odostomia* spp | 0.006 | 0.006 | 0.918 | 0.019 | 0.034 | 0.791 |  |
| *Bittium submamillatum* | 0.005 | 0.008 | 0.672 | 0.000 | 0.038 | 0.802 |  |
| *Aporrhais pespelecani* | 0.005 | 0.006 | 0.813 | 0.026 | 0.019 | 0.812 |  |
| *Lentidium mediterraneum* | 0.005 | 0.005 | 0.889 | 0.026 | 0.025 | 0.823 |  |
| *Polititapes* spp | 0.005 | 0.007 | 0.687 | 0.033 | 0.000 | 0.833 |  |
| *Veneridae* indet | 0.005 | 0.007 | 0.687 | 0.033 | 0.000 | 0.843 |  |
| *Pecten jacobaeus* | 0.004 | 0.006 | 0.674 | 0.000 | 0.031 | 0.852 |  |
| *Philine quadripartita* | 0.004 | 0.006 | 0.669 | 0.000 | 0.030 | 0.861 |  |
| *Hiatella arctica* | 0.004 | 0.005 | 0.794 | 0.019 | 0.019 | 0.870 |  |
| *Fusinus rostratus* | 0.004 | 0.006 | 0.684 | 0.000 | 0.028 | 0.879 |  |
| *Nassarius reticulatus* | 0.004 | 0.005 | 0.687 | 0.026 | 0.000 | 0.887 |  |
| *Spisula subtruncata* | 0.004 | 0.004 | 0.809 | 0.019 | 0.013 | 0.894 |  |
| Land snail 1 | 0.003 | 0.004 | 0.813 | 0.019 | 0.013 | 0.902 |  |
| *Mimachlamys varia* | 0.003 | 0.007 | 0.434 | 0.000 | 0.020 | 0.908 |  |
| *Euspira macilenta* | 0.003 | 0.007 | 0.434 | 0.000 | 0.020 | 0.914 |  |
| *Hexaplex trunculus* juv | 0.003 | 0.007 | 0.434 | 0.000 | 0.020 | 0.920 |  |
| *Anadara gibbosa* | 0.003 | 0.004 | 0.687 | 0.019 | 0.000 | 0.926 |  |
| *Monia patelliformes* | 0.003 | 0.004 | 0.687 | 0.019 | 0.000 | 0.931 |  |
| *Thyasira* spp | 0.003 | 0.004 | 0.687 | 0.019 | 0.000 | 0.937 |  |
| *Thracia corbuloides* | 0.003 | 0.004 | 0.687 | 0.019 | 0.000 | 0.942 |  |
| *Parvicardium minimum* | 0.003 | 0.006 | 0.434 | 0.000 | 0.017 | 0.948 |  |
| *Hydrobia* sp 1 cf acuta | 0.003 | 0.006 | 0.434 | 0.000 | 0.017 | 0.953 |  |
| *Akera bullata* | 0.003 | 0.006 | 0.434 | 0.000 | 0.017 | 0.959 |  |
| *Nucula sulcata* | 0.002 | 0.006 | 0.434 | 0.000 | 0.019 | 0.964 |  |
| *Musculus subpictus* | 0.002 | 0.005 | 0.434 | 0.000 | 0.015 | 0.968 |  |
| *Ostrea* spp | 0.002 | 0.005 | 0.434 | 0.000 | 0.015 | 0.973 |  |
| *Epitonium muricatum* | 0.002 | 0.005 | 0.434 | 0.000 | 0.015 | 0.978 |  |
| *Fustiaria rubescens* | 0.002 | 0.004 | 0.434 | 0.000 | 0.013 | 0.982 |  |
| *Cerastoderma edule* | 0.002 | 0.004 | 0.434 | 0.000 | 0.013 | 0.985 |  |
| *Mangelia attenuata* | 0.002 | 0.004 | 0.434 | 0.000 | 0.013 | 0.989 |  |
| *Odostomia* cf *eulimoides* | 0.002 | 0.004 | 0.434 | 0.000 | 0.013 | 0.993 |  |
| *Acteon tornatilis* | 0.002 | 0.004 | 0.434 | 0.000 | 0.013 | 0.996 |  |
| *Mysia undata* | 0.002 | 0.004 | 0.434 | 0.000 | 0.012 | 1.000 |  |
| **Station 1 – Establishment stage (group A) vs introduction stage (group B)** | | | | | | | |
| *Anadara transversa* | 0.037 | 0.008 | 4.700 | 0.362 | 0.093 | 0.094 |  |
| *Nassarius pygmaeus* | 0.029 | 0.015 | 2.005 | 0.104 | 0.316 | 0.167 |  |
| *Nucula* cf *nuclaeus* | 0.026 | 0.008 | 3.099 | 0.269 | 0.086 | 0.232 |  |
| *Bittium submamillatum* | 0.023 | 0.009 | 2.717 | 0.000 | 0.167 | 0.290 |  |
| *Aequipecten opercularis* | 0.016 | 0.010 | 1.562 | 0.033 | 0.139 | 0.330 |  |
| *Aporrhais pespelecani* | 0.014 | 0.008 | 1.786 | 0.026 | 0.125 | 0.365 |  |
| *Cylichna cylindracea* | 0.013 | 0.008 | 1.633 | 0.136 | 0.039 | 0.399 |  |
| *Saxicavella jeffreysi* | 0.013 | 0.007 | 1.719 | 0.019 | 0.103 | 0.431 |  |
| *Kurtiella bidentata* | 0.012 | 0.010 | 1.270 | 0.215 | 0.297 | 0.462 |  |
| *Pitar rudis* | 0.012 | 0.008 | 1.498 | 0.155 | 0.072 | 0.491 |  |
| *Corbula gibba* | 0.010 | 0.006 | 1.692 | 0.684 | 0.735 | 0.516 |  |
| *Abra nitida* | 0.010 | 0.004 | 2.321 | 0.166 | 0.095 | 0.541 |  |
| *Moerella cf distorta* | 0.009 | 0.005 | 2.023 | 0.119 | 0.052 | 0.564 |  |
| *Abra alba* | 0.009 | 0.006 | 1.358 | 0.074 | 0.074 | 0.586 |  |
| *Musculus subpictus* | 0.009 | 0.005 | 1.643 | 0.000 | 0.062 | 0.607 |  |
| *Antalis inaequicostata* | 0.008 | 0.006 | 1.235 | 0.061 | 0.017 | 0.627 |  |
| *Polititapes* cf *rhomboides* | 0.008 | 0.007 | 1.179 | 0.047 | 0.099 | 0.646 |  |
| *Hyala vitrea* | 0.007 | 0.006 | 1.218 | 0.146 | 0.117 | 0.665 |  |
| *Flexopecten glaber* | 0.007 | 0.006 | 1.168 | 0.145 | 0.120 | 0.683 |  |
| *Thyasira biplicata* | 0.007 | 0.006 | 1.181 | 0.060 | 0.053 | 0.700 |  |
| *Hiatella arctica* | 0.007 | 0.006 | 1.137 | 0.019 | 0.053 | 0.717 |  |
| *Acanthocardia paucicostata* | 0.006 | 0.003 | 1.925 | 0.066 | 0.066 | 0.734 |  |
| *Calyptraea chinensis* | 0.006 | 0.004 | 1.433 | 0.060 | 0.051 | 0.749 |  |
| *Mangelia costulata* | 0.006 | 0.006 | 1.002 | 0.033 | 0.028 | 0.763 |  |
| *Spisula subtruncata* | 0.006 | 0.004 | 1.298 | 0.019 | 0.051 | 0.777 |  |
| *Turritella communis* | 0.005 | 0.004 | 1.524 | 0.206 | 0.190 | 0.791 |  |
| *Anomia ephippium* | 0.005 | 0.005 | 1.016 | 0.028 | 0.024 | 0.804 |  |
| *Philine scabra* | 0.005 | 0.006 | 0.782 | 0.000 | 0.035 | 0.816 |  |
| *Polititapes* spp | 0.005 | 0.007 | 0.683 | 0.033 | 0.000 | 0.827 |  |
| *Veneridae* indet | 0.005 | 0.007 | 0.683 | 0.033 | 0.000 | 0.839 |  |
| *Nassarius lima* | 0.005 | 0.007 | 0.683 | 0.033 | 0.000 | 0.850 |  |
| *Phaxas adriaticus* | 0.005 | 0.005 | 0.826 | 0.028 | 0.011 | 0.862 |  |
| *Lentidium mediterraneum* | 0.004 | 0.005 | 0.833 | 0.026 | 0.012 | 0.872 |  |
| *Nassarius reticulatus* | 0.004 | 0.005 | 0.833 | 0.026 | 0.012 | 0.882 |  |
| *Eulima glabra* | 0.004 | 0.004 | 0.922 | 0.019 | 0.024 | 0.892 |  |
| *Mysia undata* | 0.004 | 0.005 | 0.771 | 0.000 | 0.027 | 0.901 |  |
| *Monia patelliformes* | 0.004 | 0.004 | 0.905 | 0.019 | 0.023 | 0.910 |  |
| *Anadara gibbosa* | 0.003 | 0.004 | 0.683 | 0.019 | 0.000 | 0.916 |  |
| *Thyasira* spp | 0.003 | 0.004 | 0.683 | 0.019 | 0.000 | 0.923 |  |
| *Thracia corbuloides* | 0.003 | 0.004 | 0.683 | 0.019 | 0.000 | 0.929 |  |
| *Odostomia* spp | 0.003 | 0.004 | 0.683 | 0.019 | 0.000 | 0.936 |  |
| *Gastropoda* indet | 0.003 | 0.004 | 0.683 | 0.019 | 0.000 | 0.942 |  |
| *Odostomia cf eulimoides* | 0.002 | 0.005 | 0.483 | 0.000 | 0.017 | 0.948 |  |
| *Philine quadripartita* | 0.002 | 0.005 | 0.483 | 0.000 | 0.017 | 0.954 |  |
| *Coracuta obliquata* | 0.002 | 0.005 | 0.483 | 0.000 | 0.016 | 0.959 |  |
| *Timoclea ovata* | 0.002 | 0.004 | 0.483 | 0.000 | 0.012 | 0.964 |  |
| *Bivalvia* indet | 0.002 | 0.004 | 0.483 | 0.000 | 0.012 | 0.968 |  |
| Land snail 1 | 0.002 | 0.004 | 0.483 | 0.000 | 0.012 | 0.972 |  |
| *Papillicardium papillosum* | 0.002 | 0.003 | 0.483 | 0.000 | 0.012 | 0.976 |  |
| *Ostrea* spp | 0.002 | 0.003 | 0.483 | 0.000 | 0.012 | 0.981 |  |
| *Ringicula conformis* | 0.002 | 0.003 | 0.483 | 0.000 | 0.012 | 0.985 |  |
| *Anadara inaequivalvis* | 0.002 | 0.003 | 0.483 | 0.000 | 0.011 | 0.988 |  |
| *Pectinidae* indet | 0.002 | 0.003 | 0.483 | 0.000 | 0.011 | 0.992 |  |
| *Parvicardium minimum* | 0.002 | 0.003 | 0.483 | 0.000 | 0.011 | 0.996 |  |
| *Euspira nitida* | 0.002 | 0.003 | 0.483 | 0.000 | 0.011 | 1.000 |  |
| **Station 2 – Introduction stage (group A) vs stage without *Anadara* (group B)** | | | | | | | |
| *Turritella communis* | 0.033 | 0.018 | 1.816 | 0.248 | 0.481 | 0.077 |  |
| *Corbula gibba* | 0.027 | 0.011 | 2.497 | 0.738 | 0.541 | 0.140 |  |
| *Nassarius pygmaeus* | 0.021 | 0.014 | 1.493 | 0.276 | 0.131 | 0.189 |  |
| *Nucula* cf *nuclaeus* | 0.018 | 0.009 | 1.941 | 0.116 | 0.248 | 0.231 |  |
| *Abra nitida* | 0.017 | 0.007 | 2.253 | 0.031 | 0.154 | 0.270 |  |
| *Acanthocardia paucicostata* | 0.014 | 0.009 | 1.558 | 0.093 | 0.188 | 0.303 |  |
| *Flexopecten glaber* | 0.013 | 0.006 | 2.271 | 0.113 | 0.020 | 0.335 |  |
| *Aequipecten opercularis* | 0.012 | 0.010 | 1.300 | 0.105 | 0.115 | 0.364 |  |
| *Anadara transversa* | 0.012 | 0.007 | 1.642 | 0.086 | 0.000 | 0.392 |  |
| *Kurtiella bidentata* | 0.012 | 0.007 | 1.613 | 0.286 | 0.335 | 0.420 |  |
| *Cylichna cylindracea* | 0.010 | 0.005 | 2.233 | 0.084 | 0.020 | 0.443 |  |
| *Hiatella arctica* | 0.010 | 0.007 | 1.371 | 0.083 | 0.055 | 0.466 |  |
| *Eulima glabra* | 0.010 | 0.008 | 1.181 | 0.016 | 0.074 | 0.489 |  |
| *Calyptraea chinensis* | 0.010 | 0.008 | 1.131 | 0.023 | 0.072 | 0.511 |  |
| *Coracuta obliquata* | 0.009 | 0.008 | 1.168 | 0.000 | 0.070 | 0.533 |  |
| *Moerella* cf *distorta* | 0.009 | 0.006 | 1.362 | 0.062 | 0.073 | 0.553 |  |
| *Thyasira biplicata* | 0.008 | 0.005 | 1.845 | 0.061 | 0.023 | 0.572 |  |
| *Bittium submamillatum* | 0.008 | 0.006 | 1.446 | 0.069 | 0.016 | 0.592 |  |
| *Abra prismatica* | 0.008 | 0.006 | 1.200 | 0.016 | 0.062 | 0.610 |  |
| *Aporrhais pespelecani* | 0.008 | 0.007 | 1.186 | 0.037 | 0.062 | 0.628 |  |
| *Retusa laevisculpta* | 0.008 | 0.006 | 1.217 | 0.053 | 0.065 | 0.645 |  |
| *Euspira macilenta* | 0.008 | 0.005 | 1.585 | 0.061 | 0.067 | 0.663 |  |
| *Antalis inaequicostata* | 0.008 | 0.007 | 1.122 | 0.054 | 0.000 | 0.681 |  |
| *Veneridae* indet | 0.007 | 0.007 | 1.110 | 0.052 | 0.000 | 0.698 |  |
| *Phaxas adriaticus* | 0.007 | 0.006 | 1.196 | 0.000 | 0.055 | 0.715 |  |
| *Lentidium mediterraneum* | 0.007 | 0.006 | 1.207 | 0.031 | 0.059 | 0.732 |  |
| *Mangelia costulata* | 0.007 | 0.005 | 1.204 | 0.047 | 0.020 | 0.747 |  |
| *Odostomia* spp | 0.006 | 0.007 | 0.883 | 0.016 | 0.044 | 0.762 |  |
| *Abra alba* | 0.006 | 0.008 | 0.783 | 0.000 | 0.047 | 0.776 |  |
| *Pitar rudis* | 0.006 | 0.004 | 1.439 | 0.130 | 0.133 | 0.790 |  |
| *Hyala vitrea* | 0.006 | 0.006 | 0.883 | 0.036 | 0.020 | 0.803 |  |
| *Ensis minor* | 0.006 | 0.007 | 0.795 | 0.000 | 0.042 | 0.816 |  |
| *Musculus subpictus* | 0.006 | 0.007 | 0.794 | 0.000 | 0.042 | 0.829 |  |
| *Philine quadripartita* | 0.005 | 0.006 | 0.886 | 0.016 | 0.035 | 0.841 |  |
| *Polititapes* cf *rhomboides* | 0.005 | 0.006 | 0.922 | 0.031 | 0.020 | 0.853 |  |
| *Euspira nitida* | 0.005 | 0.007 | 0.769 | 0.037 | 0.000 | 0.865 |  |
| *Anomia ephippium* | 0.004 | 0.005 | 0.791 | 0.031 | 0.000 | 0.875 |  |
| *Episiphon filum* | 0.004 | 0.009 | 0.490 | 0.000 | 0.032 | 0.885 |  |
| *Acteon tornatilis* | 0.004 | 0.005 | 0.795 | 0.030 | 0.000 | 0.894 |  |
| *Saxicavella jeffreysi* | 0.004 | 0.003 | 1.221 | 0.169 | 0.175 | 0.904 |  |
| *Hemilepton nitidum* | 0.004 | 0.005 | 0.696 | 0.014 | 0.020 | 0.912 |  |
| *Ringicula conformis* | 0.004 | 0.005 | 0.681 | 0.015 | 0.016 | 0.921 |  |
| *Azorinus chamasolen* | 0.003 | 0.006 | 0.490 | 0.000 | 0.023 | 0.928 |  |
| *Philine scabra* | 0.003 | 0.005 | 0.490 | 0.000 | 0.019 | 0.934 |  |
| *Tapetinae* indet | 0.003 | 0.005 | 0.490 | 0.000 | 0.020 | 0.939 |  |
| *Mangelia attenuata* | 0.002 | 0.005 | 0.488 | 0.016 | 0.000 | 0.945 |  |
| *Venus casina* | 0.002 | 0.005 | 0.490 | 0.000 | 0.016 | 0.950 |  |
| *Moerella* indet | 0.002 | 0.005 | 0.488 | 0.016 | 0.000 | 0.955 |  |
| *Alvania geryonia* | 0.002 | 0.005 | 0.488 | 0.016 | 0.000 | 0.961 |  |
| *Alvania lineata* | 0.002 | 0.005 | 0.488 | 0.016 | 0.000 | 0.966 |  |
| *Ostrea* spp | 0.002 | 0.004 | 0.488 | 0.016 | 0.000 | 0.971 |  |
| *Viviparus* sp 1 | 0.002 | 0.004 | 0.488 | 0.016 | 0.000 | 0.976 |  |
| *Loripes lacteus* | 0.002 | 0.004 | 0.488 | 0.016 | 0.000 | 0.981 |  |
| *Nassarius reticulatus* | 0.002 | 0.004 | 0.488 | 0.015 | 0.000 | 0.986 |  |
| *Polititapes* spp | 0.002 | 0.004 | 0.488 | 0.015 | 0.000 | 0.991 |  |
| *Bela brachystoma* | 0.002 | 0.004 | 0.488 | 0.015 | 0.000 | 0.995 |  |
| *Mysia undata* | 0.002 | 0.004 | 0.488 | 0.014 | 0.000 | 1.000 |  |
| **Station 2 – Establishment stage (group A) vs stage without *Anadara* (group B)** | | | | | | | |
| *Turritella communis* | 0.060 | 0.019 | 3.192 | 0.070 | 0.481 | 0.124 |  |
| *Anadara transversa* | 0.037 | 0.003 | 10.684 | 0.256 | 0.000 | 0.201 |  |
| *Corbula gibba* | 0.025 | 0.013 | 1.938 | 0.708 | 0.541 | 0.253 |  |
| *Flexopecten glaber* | 0.024 | 0.007 | 3.518 | 0.185 | 0.020 | 0.303 |  |
| *Acanthocardia paucicostata* | 0.019 | 0.011 | 1.736 | 0.065 | 0.188 | 0.342 |  |
| *Nucula* cf *nuclaeus* | 0.017 | 0.015 | 1.180 | 0.136 | 0.248 | 0.378 |  |
| *Abra nitida* | 0.017 | 0.008 | 2.049 | 0.073 | 0.154 | 0.413 |  |
| *Nassarius pygmaeus* | 0.015 | 0.012 | 1.266 | 0.212 | 0.131 | 0.445 |  |
| *Saxicavella jeffreysi* | 0.011 | 0.011 | 0.999 | 0.244 | 0.175 | 0.468 |  |
| *Aequipecten opercularis* | 0.011 | 0.008 | 1.342 | 0.081 | 0.115 | 0.490 |  |
| *Eulima glabra* | 0.010 | 0.009 | 1.132 | 0.000 | 0.074 | 0.512 |  |
| *Calyptraea chinensis* | 0.010 | 0.009 | 1.097 | 0.000 | 0.072 | 0.533 |  |
| *Euspira macilenta* | 0.010 | 0.008 | 1.148 | 0.000 | 0.067 | 0.553 |  |
| *Coracuta obliquata* | 0.009 | 0.008 | 1.161 | 0.000 | 0.070 | 0.573 |  |
| *Moerella* cf *distorta* | 0.009 | 0.007 | 1.267 | 0.063 | 0.073 | 0.593 |  |
| *Pitar rudis* | 0.009 | 0.007 | 1.272 | 0.095 | 0.133 | 0.612 |  |
| *Anomia ephippium* | 0.009 | 0.005 | 1.634 | 0.062 | 0.000 | 0.631 |  |
| *Thyasira biplicata* | 0.009 | 0.006 | 1.484 | 0.065 | 0.023 | 0.649 |  |
| *Retusa laevisculpta* | 0.008 | 0.006 | 1.374 | 0.038 | 0.065 | 0.666 |  |
| *Lentidium mediterraneum* | 0.008 | 0.007 | 1.173 | 0.000 | 0.059 | 0.683 |  |
| *Hyala vitrea* | 0.008 | 0.008 | 1.005 | 0.056 | 0.020 | 0.700 |  |
| *Aporrhais pespelecani* | 0.008 | 0.007 | 1.104 | 0.025 | 0.062 | 0.717 |  |
| *Abra prismatica* | 0.008 | 0.007 | 1.049 | 0.052 | 0.062 | 0.733 |  |
| *Antalis inaequicostata* | 0.008 | 0.008 | 0.910 | 0.054 | 0.000 | 0.749 |  |
| *Musculus subpictus* | 0.007 | 0.007 | 1.082 | 0.045 | 0.042 | 0.764 |  |
| *Hiatella arctica* | 0.007 | 0.006 | 1.214 | 0.017 | 0.055 | 0.779 |  |
| *Phaxas adriaticus* | 0.007 | 0.006 | 1.178 | 0.019 | 0.055 | 0.794 |  |
| *Polititapes* cf *rhomboides* | 0.007 | 0.007 | 1.001 | 0.045 | 0.020 | 0.809 |  |
| *Cylichna cylindracea* | 0.006 | 0.006 | 1.005 | 0.043 | 0.020 | 0.822 |  |
| *Abra alba* | 0.006 | 0.008 | 0.779 | 0.000 | 0.047 | 0.835 |  |
| *Odostomia* spp | 0.006 | 0.008 | 0.768 | 0.000 | 0.044 | 0.848 |  |
| *Kurtiella bidentata* | 0.006 | 0.004 | 1.667 | 0.342 | 0.335 | 0.860 |  |
| *Ensis minor* | 0.006 | 0.007 | 0.791 | 0.000 | 0.042 | 0.872 |  |
| *Philine quadripartita* | 0.006 | 0.006 | 0.933 | 0.018 | 0.035 | 0.884 |  |
| *Pectinidae* indet | 0.005 | 0.010 | 0.561 | 0.039 | 0.000 | 0.896 |  |
| *Bittium submamillatum* | 0.005 | 0.007 | 0.740 | 0.027 | 0.016 | 0.906 |  |
| *Episiphon filum* | 0.004 | 0.009 | 0.487 | 0.000 | 0.032 | 0.916 |  |
| *Limatula subauriculata* | 0.004 | 0.007 | 0.561 | 0.028 | 0.000 | 0.924 |  |
| *Cerastoderma* spp | 0.004 | 0.007 | 0.561 | 0.028 | 0.000 | 0.932 |  |
| *Triphoridae* indet | 0.004 | 0.007 | 0.561 | 0.028 | 0.000 | 0.940 |  |
| *Azorinus chamasolen* | 0.003 | 0.006 | 0.487 | 0.000 | 0.023 | 0.946 |  |
| *Mimachlamys varia* | 0.003 | 0.005 | 0.560 | 0.019 | 0.000 | 0.952 |  |
| *Atys jeffreysi* | 0.003 | 0.005 | 0.560 | 0.019 | 0.000 | 0.958 |  |
| *Philine scabra* | 0.003 | 0.005 | 0.487 | 0.000 | 0.019 | 0.964 |  |
| *Hemilepton nitidum* | 0.003 | 0.005 | 0.487 | 0.000 | 0.020 | 0.969 |  |
| *Mangelia costulata* | 0.003 | 0.005 | 0.487 | 0.000 | 0.020 | 0.975 |  |
| *Tapetinae* indet | 0.003 | 0.005 | 0.487 | 0.000 | 0.020 | 0.980 |  |
| *Anadara inaequivalvis* | 0.002 | 0.004 | 0.561 | 0.017 | 0.000 | 0.985 |  |
| *Spisula subtruncata* | 0.002 | 0.004 | 0.561 | 0.017 | 0.000 | 0.990 |  |
| *Ringicula conformis* | 0.002 | 0.005 | 0.487 | 0.000 | 0.016 | 0.995 |  |
| *Venus casina* | 0.002 | 0.005 | 0.487 | 0.000 | 0.016 | 1.000 |  |
| **Station 2 – Establishment stage (group A) vs introduction stage** | | | | | | | |
| *Turritella communis* | 0.026 | 0.015 | 1.692 | 0.070 | 0.248 | 0.069 |  |
| *Anadara transversa* | 0.025 | 0.007 | 3.358 | 0.256 | 0.086 | 0.136 |  |
| *Nucula* cf *nuclaeus* | 0.013 | 0.008 | 1.762 | 0.136 | 0.116 | 0.173 |  |
| *Aequipecten opercularis* | 0.013 | 0.009 | 1.384 | 0.081 | 0.105 | 0.208 |  |
| *Acanthocardia paucicostata* | 0.012 | 0.009 | 1.319 | 0.065 | 0.093 | 0.242 |  |
| *Kurtiella bidentata* | 0.012 | 0.007 | 1.857 | 0.342 | 0.286 | 0.275 |  |
| *Saxicavella jeffreysi* | 0.012 | 0.012 | 1.003 | 0.244 | 0.169 | 0.307 |  |
| *Hiatella arctica* | 0.011 | 0.009 | 1.267 | 0.017 | 0.083 | 0.338 |  |
| *Abra nitida* | 0.011 | 0.011 | 0.949 | 0.073 | 0.031 | 0.367 |  |
| *Flexopecten glaber* | 0.011 | 0.004 | 2.709 | 0.185 | 0.113 | 0.395 |  |
| *Nassarius pygmaeus* | 0.010 | 0.009 | 1.104 | 0.212 | 0.276 | 0.423 |  |
| *Pitar rudis* | 0.010 | 0.007 | 1.332 | 0.095 | 0.130 | 0.449 |  |
| *Bittium submamillatum* | 0.009 | 0.006 | 1.571 | 0.027 | 0.069 | 0.474 |  |
| *Euspira macilenta* | 0.009 | 0.005 | 1.936 | 0.000 | 0.061 | 0.498 |  |
| *Corbula gibba* | 0.009 | 0.007 | 1.181 | 0.708 | 0.738 | 0.521 |  |
| *Antalis inaequicostata* | 0.008 | 0.007 | 1.230 | 0.054 | 0.054 | 0.544 |  |
| *Hyala vitrea* | 0.008 | 0.007 | 1.099 | 0.056 | 0.036 | 0.566 |  |
| *Veneridae* indet | 0.008 | 0.007 | 1.110 | 0.000 | 0.052 | 0.588 |  |
| *Abra prismatica* | 0.008 | 0.007 | 1.049 | 0.052 | 0.016 | 0.608 |  |
| *Cylichna cylindracea* | 0.007 | 0.006 | 1.308 | 0.043 | 0.084 | 0.628 |  |
| *Anomia ephippium* | 0.007 | 0.006 | 1.233 | 0.062 | 0.031 | 0.647 |  |
| *Mangelia costulata* | 0.007 | 0.006 | 1.185 | 0.000 | 0.047 | 0.666 |  |
| *Polititapes* cf *rhomboides* | 0.007 | 0.006 | 1.075 | 0.045 | 0.031 | 0.684 |  |
| *Retusa laevisculpta* | 0.007 | 0.006 | 1.145 | 0.038 | 0.053 | 0.702 |  |
| *Aporrhais pespelecani* | 0.006 | 0.007 | 0.927 | 0.025 | 0.037 | 0.719 |  |
| *Musculus subpictus* | 0.006 | 0.007 | 0.926 | 0.045 | 0.000 | 0.736 |  |
| *Pectinidae* indet | 0.006 | 0.010 | 0.563 | 0.039 | 0.000 | 0.751 |  |
| *Moerella* cf *distorta* | 0.005 | 0.005 | 1.060 | 0.063 | 0.062 | 0.766 |  |
| *Thyasira biplicata* | 0.005 | 0.005 | 1.028 | 0.065 | 0.061 | 0.781 |  |
| *Euspira nitida* | 0.005 | 0.007 | 0.768 | 0.000 | 0.037 | 0.795 |  |
| *Lentidium mediterraneum* | 0.005 | 0.006 | 0.790 | 0.000 | 0.031 | 0.807 |  |
| *Acteon tornatilis* | 0.004 | 0.005 | 0.794 | 0.000 | 0.030 | 0.819 |  |
| *Philine quadripartita* | 0.004 | 0.006 | 0.730 | 0.018 | 0.016 | 0.830 |  |
| *Limatula subauriculata* | 0.004 | 0.007 | 0.563 | 0.028 | 0.000 | 0.841 |  |
| *Cerastoderma* spp | 0.004 | 0.007 | 0.563 | 0.028 | 0.000 | 0.851 |  |
| *Triphoridae* indet | 0.004 | 0.007 | 0.563 | 0.028 | 0.000 | 0.862 |  |
| *Calyptraea chinensis* | 0.003 | 0.007 | 0.487 | 0.000 | 0.023 | 0.871 |  |
| *Mimachlamys varia* | 0.003 | 0.005 | 0.563 | 0.019 | 0.000 | 0.879 |  |
| *Phaxas adriaticus* | 0.003 | 0.005 | 0.563 | 0.019 | 0.000 | 0.887 |  |
| *Atys jeffreysi* | 0.003 | 0.005 | 0.563 | 0.019 | 0.000 | 0.895 |  |
| *Mangelia attenuata* | 0.002 | 0.005 | 0.487 | 0.000 | 0.016 | 0.901 |  |
| *Anadara inaequivalvis* | 0.002 | 0.004 | 0.563 | 0.017 | 0.000 | 0.908 |  |
| *Spisula subtruncata* | 0.002 | 0.004 | 0.563 | 0.017 | 0.000 | 0.915 |  |
| *Eulima glabra* | 0.002 | 0.005 | 0.487 | 0.000 | 0.016 | 0.921 |  |
| *Moerella* indet | 0.002 | 0.005 | 0.487 | 0.000 | 0.016 | 0.928 |  |
| *Alvania geryonia* | 0.002 | 0.005 | 0.487 | 0.000 | 0.016 | 0.934 |  |
| *Alvania lineata* | 0.002 | 0.005 | 0.487 | 0.000 | 0.016 | 0.940 |  |
| *Odostomia* spp | 0.002 | 0.005 | 0.487 | 0.000 | 0.016 | 0.946 |  |
| *Ostrea* spp | 0.002 | 0.005 | 0.487 | 0.000 | 0.016 | 0.953 |  |
| *Viviparus* sp 1 | 0.002 | 0.005 | 0.487 | 0.000 | 0.016 | 0.959 |  |
| *Loripes lacteus* | 0.002 | 0.005 | 0.487 | 0.000 | 0.016 | 0.965 |  |
| *Nassarius reticulatus* | 0.002 | 0.005 | 0.487 | 0.000 | 0.015 | 0.971 |  |
| *Ringicula conformis* | 0.002 | 0.005 | 0.487 | 0.000 | 0.015 | 0.977 |  |
| *Polititapes* spp | 0.002 | 0.005 | 0.487 | 0.000 | 0.015 | 0.983 |  |
| *Bela brachystoma* | 0.002 | 0.005 | 0.487 | 0.000 | 0.015 | 0.989 |  |
| *Hemilepton nitidum* | 0.002 | 0.004 | 0.487 | 0.000 | 0.014 | 0.994 |  |
| *Mysia undata* | 0.002 | 0.004 | 0.487 | 0.000 | 0.014 | 1.000 |  |

**Table S3**. Results of the Spearman rank-order test. Hypoxic events were extracted from Figure 2 of Djakovac et al (2015; stations SJ101 and SJ108 correspond to our stations 2 and 1, respectively) for 1972 – 2012 and from Justić (1991) from 1911 and 1972 (when no hypoxic events with DO lower than 2 mg/l were reported). Bold green highlights significant *p*-values.

|  | Median size | | Abundance | |
| --- | --- | --- | --- | --- |
|  | ρ | *p* | ρ | *p* |
| ***Anadara transversa*** | | | | |
| Hypoxic events Po4 | 0.216 | 0.608 | 0.63 | **0.016** |
| Hypoxic events Po3 | 0.327 | 0.429 | 0.571 | **0.033** |
| ***Corbula gibba*** | | | | |
| Hypoxic events Po4 | 0.831 | **0** | 0.815 | **0** |
| Hypoxic events Po3 | 0.848 | **0** | 0.715 | **0.004** |

# References

Djakovac T, Supić N, Bernardi Aubry F, Degobbis D, Giani M (2015) Mechanisms of hypoxia frequency changes in the northern Adriatic Sea during the period 1972–2012. *Journal of Marine Systems* 141:179–189.

Fossato VU, Campesan G, Craboledda L, Dolci F, Stocco G (1996) Organic micropollutants and trace metals in water and suspended particulate matter, in: Lasserre P & Marzollo A (Eds) *Venice Lagoon Ecosystem*, Unesco/Murst, Vol. 1, 8 pp.

Fossato VU, Campesan G, Dolci F, Stocco G (1998) Trends in chlorinated hydrocarbons and heavy metals in sediments of Venetian canals. *Rapports et Proces Verbaux des Reunions - Commission Internationale pour l'Exploration Scientifique de la Mer Mediterranee*. 35:258–259.

IAEA (2001) World-wide and regional intercomparison for the determination of organochlorine compounds, petroleum hydrocarbons and sterols in sediment sample. *International Atomic Energy Agency I.A.E.A.*. 417.

IAEA (2007) World-wide and regional intercomparison for the determination of organochlorine compounds, petroleum hydrocarbons and sterols in sediment sample. *International Atomic Energy Agency I.A.E.A.*. 159.

IAEA (2012) World-wide and regional intercomparison on the determination of organochlorine compounds, polybrominated diphenyl ethers and petroleum hydrocarbons in sediment sample. *International Atomic Energy Agency I.A.E.A.*. 459.

Justić D (1991) Hypoxic conditions in the northern Adriatic Sea: historical development and ecological significance. *Geological Society Special Publication*, 58:95–105.

USEPA (Environment Protection Agency of United State of America) (1994a) Method 3051A, Microwave assisted acid digestion of sediments, sludges, soils and oils.

USEPA (Environment Protection Agency of United State of America) (1994b) Method 200.7, Determination of Metals and Trace Elements in water and Wastes by Inductively Coupled Plasma-Atomic Emission Spectrometry.

USEPA (Environment Protection Agency of United State of America) (1976) Method 245.1, Mercury (Manual Cold Vapor Technique).
